# Antibacterial activity of gold nanoparticles and their toxicity assessment

**DOI:** 10.1186/1471-2334-14-S3-P64

**Published:** 2014-05-27

**Authors:** K Umamaheswari, R Baskar, K Chandru, N Rajendiran, S Chandirasekar

**Affiliations:** 1 Department of Biotechnology, University of Madras, Guindy, Chennai, India; 2 Department of Polymer Science, University of Madras, Guindy, Chennai, India; 3 Centre for Biotechnology, Anna University, Guindy, Chennai, India

## Background

Several classes of antimicrobial nanoparticles (NPs) and nanosized carriers for antibiotics delivery have proved their effectiveness as alternative agents to combat the antimicrobial resistance experienced with conventional drugs. Gold nanostructures find extensive applications in nano electronics and nanomedicine.

## Methods

The present study involved the synthesis of gold nanoparticles using a sodium cholate, as both reducing and capping agent and characterization by UV- Visible spectroscopy and High ResolutionTransmission Electron Microscopy (HRTEM). The antibacterial activity on significant bacterial species was evaluated by micro broth dilution method. The mechanism for antibacterial action of the gold nanoparticles were studied by reactive oxygen species (ROS) generation that causes oxidative stress to microbial cells and release of intracellular enzyme lactate dehydrogenase into extracellular medium (LDH assay) indicative of loss of cell membrane integrity.

## Results

The synthesized gold nanoparticles showed characteristic peak at 541 nm by UV- Visible spectroscopy with particle size of 10nm as confirmed by HRTEM. The minimum inhibitory concentration (MIC_80_) of the nanoparticles on *E. coli*, *S. typhi*, *P. aeruginosa*, *K. pneumoniae*, ranged from 20 to 40µg/mL. The ROS generation was directly dependent on the concentration of the nanoparticles and no detectable enzyme leakage was recorded.

## Conclusion

The results showed appreciable antibacterial activity of gold nanoparticles against the tested species and the possible mechanism of antibacterial activity may be due to increased intracellular ROS generation causing oxidative stress to the bacterial cells while LDH assay indicated that nanoparticles caused no damage to the cell membrane integrity.

